# Retrospective Review of 2851 Female Patients With Telogen Effluvium: A Single‐Center Experience

**DOI:** 10.1111/jocd.70037

**Published:** 2025-02-14

**Authors:** Ömer Karakoyun, Erhan Ayhan, İsmail Yıldız

**Affiliations:** ^1^ Department of Dermatology Dicle University Faculty of Medicine Diyarbakır Turkey; ^2^ Department of Biostatistics Dicle University Faculty of Medicine Diyarbakır Turkey

**Keywords:** etiology, hair loss, laboratory, telogen effluvium

## Abstract

**Objective:**

Telogen effluvium, the most common cause of diffuse and non‐scarring alopecia in women, is frequently diagnosed in dermatology outpatient clinics and can be caused by various etiological factors such as medications, trauma, emotional, and physiological stress. In patients presenting with complaints of telogen effluvium, we aimed to investigate abnormal levels of serum ferritin, vitamin B12, folic acid, thyroid function tests, and hemoglobin and determine which of these abnormal values might be significantly associated with telogen effluvium.

**Material and Methods:**

In this study, 2851 female patients diagnosed with telogen effluvium who applied to the Dicle University Dermatology outpatient clinic between January 1, 2010 and June 30, 2024 were retrospectively evaluated. The relationship between these laboratory abnormalities and telogen effluvium, as well as their significance in different age groups, was analyzed using the SPSS‐27.0 program. Associations were analyzed using Kolmogorov–Smirnov test, dependent *t*‐test, Wilcoxon test, Pearson chi‐square (*χ* ^2^) test, Yates chi‐square (*χ* ^2^) test, Fisher chi‐square (*χ* ^2^) test analysis, Mc‐Nemar test, Pearson/spearman correlation analysis, and logistic regression analysis. A *p* value of < 0.05 was considered statistically significant.

**Results:**

The mean age of 2851 female patients included in our study was 26.33 years, 12.8% (*n* = 366) were under 18 years of age, 83.5% (*n* = 2380) were between 18 and 45 years of age, and 3.7% (*n* = 105) were over 45 years of age. Hemoglobin values were determined in 2496 of 2851 patients and values below the reference range were found in 317 patients (11.1%). Ferritin was found to be low in 1123 (46.5%) of 2413 patients. Vitamin 12 deficiency was detected in 150 (5.8%), folic acid deficiency in 8 (0.6%), and iron deficiency in 685 (29.5%) patients. As can be seen in our study, deficiency of laboratory parameters, especially ferritin and serum iron, is a common finding in female patients presenting with TE.

**Conclusion:**

This study showed that biochemical tests, complete blood counts, and hormonal tests are important tools in investigating the etiology and guiding the treatment in patients diagnosed with telogen effluvium, as various etiological factors may be present. Furthermore, it was determined that parameters such as vitamin B12, ferritin, TSH, and T3 hold varying levels of importance during significant phases of women's physiological development, such as adolescence and post‐menopausal periods.

## 1 Introduction

Hair loss is a common condition, and telogen effluvium (TE), which is the most frequent cause of diffuse, non‐scarring alopecia in women, is a condition commonly diagnosed in dermatology clinics and may be associated with numerous etiological factors [1]. The normal hair cycle consists of three phases: anagen, catagen, and telogen, with the majority of hair being in the anagen phase. Telogen effluvium, caused by various etiological factors, is an abnormal condition of the hair cycle in which many hair follicles in the anagen phase prematurely transition to the telogen phase, resulting in excessive shedding of telogen hairs, leading to diffuse, non‐scarring hair loss on the scalp [2, 3]. While a daily hair loss of 100 hairs is considered normal, this number can reach up to 400 in patients with TE. This process can be classified as acute or chronic TE, depending on whether it lasts for more or < 6 months. While etiological causes may be found in acute TE, they are often unclear in chronic cases [4]. Various etiological factors, such as normal human physiology (e.g., postpartum), medications, severe infections, thyroid dysfunction, anemia, trauma, and emotional and physiological stress, can be observed in TE. In this study, we aimed to retrospectively examine the laboratory values of female patients diagnosed with TE and determine whether abnormalities in these values have a significant relationship with TE, and to identify in which age ranges TE is more common and which etiological factor predominates in these age ranges.

## 2 Material and Methods

In this study, the laboratory values of 2851 female patients aged between 8 and 79 years, who were diagnosed with TE and presented to the Dicle University Dermatology outpatient clinic between January 1, 2010 and June 30, 2024, were retrospectively evaluated. Patients with missing or incomplete laboratory results were excluded from the study, and the results of the remaining patients were analyzed. The data were analyzed using the SPSS‐27.0 statistical program, and the value, mean, median, standard deviation, and frequency of each parameter in the total patient population were recorded. Associations were analyzed using Kolmogorov–Smirnov test, dependent *t*‐test, Wilcoxon test, Pearson chi‐square (*χ* ^2^) test, Yates chi‐square (*χ* ^2^) test, Fisher chi‐square (*χ* ^2^) test analysis, Mc‐Nemar test, Pearson/spearman correlation analysis, and logistic regression analysis. A *p* value of < 0.05 was considered statistically significant.

## 3 Results

All the patients included in our study were female (100%). The ages of the 2851 patients ranged from 8 to 79, with a mean age of 26.33. Of these, 12.8% (*n* = 366) were under the age of 18, 83.5% (*n* = 2380) were between 18 and 45 years old, and 3.7% (*n* = 105) were over the age of 45. The biochemical and hematological values identified during the study are presented in Table 1. Hemoglobin levels were evaluated in 2496 patients, and 317 patients (11.1%) were found to have values below the reference range. Low ferritin levels were detected in 1123 of 2413 patients (46.5%). Vitamin B12 deficiency was identified in 150 patients (5.8%), folic acid deficiency in 8 patients (0.6%), and iron deficiency in 685 patients (29.5%). When patients were divided into three groups according to adolescence, reproductive age, and post‐menopausal periods, the data were classified in Table 2. In our study, ferritin levels were not elevated in any of the 2413 patients, while 1123 patients (46.5%) were found to have ferritin deficiency. It was found that 961 patients (85.6%) were between the ages of 18 and 45, 127 patients (11.3%) were under the age of 18, and 35 patients (3.1%) were over the age of 45. Ferritin deficiency was predominantly observed in the 18–45 age group, and similar to previous literature, a significant relationship (*p* = 0.041) was found.

**TABLE 1:** Distribution of laboratory parameters according to reference ranges.

Percentage of laboratory parameters in 2851 patients (%)	Number of patients tested (*n*)	Reference range	Mean value	Below reference (*n*/%)	Above reference (*n*/%)
Hb (87.55%)	2496	12–16 g/dL	13.40	317 (11.1%)	121 (4.8%)
Ferritin (84.64%)	2413	18–370 ng/dL	29.69	1123 (46.5%)	0 (0%)
Iron (81.41%)	2321	60–180 μg/dL	241.31	685 (29.5%)	541 (23.3%)
Vitamin B12 (90.74%)	2587	160–900 pg/mL	376.72	150 (5.8%)	74 (2.9%)
Folic acid (50.16%)	1430	3.0–17 ng/mL	8.62	8 (0.6%)	40 (2.8%)
TSH (91.41%)	2606	0.35–4.94 mIU/L	1.98	29 (1%.1)	63 (2.4%)
T3 (35.99%)	1026	2.5–5 ng/dL	4.45	13 (1%.3)	314 (30.6%)

**TABLE 2:** Distribution of laboratory parameters according to reference values and age scale.

Parameters	Reference ranges	Under 18 years old	18–45 years old	Over 45 years	*p*
Hb *n* = 2496	< 12 g/dL	*n* = 36 (11.07%)	*n* = 263 (12.63%)	*n* = 18 (20.2)	0.2268
	12–16 g/dL	*n* = 275 (84.61%)	*n* = 1716 (82.42%)	*n* = 67 (75.2%)	
	> 16 g/dL	*n* = 14 (4.30%)	*n* = 103 (4.94%)	*n* = 4 (4494%)	
Ferritin *n* = 2413	< 18 ng/dL	*n* = 127 (40.18%)	*n* = 961 (47.64%)	*n* = 35 (43.7%)	0.0415
	18–370 ng/dL	*n* = 189 (59.81%)	*n* = 1056 (52.35%)	*n* = 45 (56.25%)	
	> 370 ng/dL	*n* = 0 (0%)	*n* = 0 (0%)	*n* = 0 (0%)	
Iron *n* = 2321	< 60 μg/dL	*n* = 84 (27.36%)	*n* = 573 (29.73%)	*n* = 28 (32.18%)	0.2906
	60–180 μg/dL	*n* = 143 (46.57%)	*n* = 906 (47.01%)	*n* = 46 (52.87%)	
	> 180 μg/dL	*n* = 80 (26.05%)	*n* = 448 (23.24%)	*n* = 13 (14.9%)	
Vitamin B12 *n* = 2587	< 160 pg/mL	*n* = 14 (4.19%)	*n* = 131 (6.07%)	*n* = 5 (5.10%)	0.0315
	160–900 pg/mL	*n* = 302 (90.41%)	*n* = 1971 (91.46%)	*n* = 90 (91.83%)	
	> 900 pg/mL	*n* = 18 (5.38%)	*n* = 53 (2459%)	*n* = 3 (3.06%)	
Folic acid *n* = 1430	< 3.0 ng/mL	*n* = 0 (0%)	*n* = 7 (0.58%)	*n* = 1 (1.58%)	0.6035
	3.0–17 ng/mL	*n* = 174 (96.6%)	*n* = 1147 (96.6%)	*n* = 61 (96.82%)	
	> 17 ng/mL	*n* = 6 (3.3%)	*n* = 33 (2.78%)	*n* = 1 (1.58%)	
TSH *n* = 2606	< 0.35 mIU/L	*n* = 1 (0.30%)	*n* = 25 (1.14%)	*n* = 3 (3.15%)	0.1015
	0.35–4.94 mIU/L	*n* = 317 (96.64%)	*n* = 2109 (96.6%)	*n* = 88 (92.6%)	
	> 4.94 mIU/L	*n* = 10 (3.0%)	*n* = 49 (2.24%)	*n* = 4 (4.21%)	
T3 *n* = 1026	< 2.5 ng/dL	*n* = 0 (0%)	*n* = 13 (1.51%)	*n* = 0 (0%)	< 0.001
	2.5–5 ng/dL	*n* = 62 (49.2%)	*n* = 605 (70.43%)	*n* = 32 (78.04%)	
	> 5 ng/dL	*n* = 64 (50.79%)	*n* = 241 (28.0%)	*n* = 9 (21.9%)	

Serum iron levels were examined in 2321 patients, and 685 (29.5%) were found to have low levels. Of these 685 patients, 84 (12.3%) were under the age of 18, 573 (83.6%) were between the ages of 18 and 45, and 28 (4.1%) were over the age of 45. No significant relationship was found between serum iron levels and age groups (*p* = 0.290). Vitamin B12 levels were found to be elevated in 74 patients (2.9%) and deficient in 150 patients (5.8%) out of 2587 patients. Of these 150 patients, 14 (9.3%) were under the age of 18, 131 (87.3%) were in the 18–45 age group, and 5 (3.3%) were over the age of 45. As can be seen from this statistic, a significant relationship was found between TE with vitamin B12 deficiency and age groups (*p* = 0.031). Folic acid levels were elevated in 40 patients (2.8%) and deficient in 8 patients (0.6%) out of 1430 patients. Of these 8 patients, 7 (87.5%) were between the ages of 18 and 45, and only 1 (12.5%) was over the age of 45. No statistically significant difference was found between the groups regarding folic acid deficiency (*p* = 0.603). TSH levels were tested in 2606 TE patients, and TSH deficiency was detected in 29 of them. Of these 29 patients, 25 (86.2%) were between the ages of 18 and 45. TSH elevation was detected in 63 patients (2.4%), with 49 of these patients (77.8%) being in the 18–45 age group. T3 levels were examined in 1026 patients, and deficiency was found in 13 patients (1.3%), all of whom (100%) were in the 18–45 age group. T3 elevation was found in 314 patients (30.6%), with 241 (76.8%) of these 314 patients being in the 18–45 age group. As can be understood from these statistics, a significant relationship was found between TE associated with thyroid dysfunction and T3 levels (*p* < 0.001), whereas no significant relationship was found with TSH levels (*p* = 0.101). Hemoglobin levels were examined in 2496 TE patients, and 317 (12.7%) were found to have low hemoglobin levels, with 263 (83%) of them in the 18–45 age group. As can be seen from these statistics, no significant relationship was found between TE caused by low hemoglobin levels and age groups (*p* = 0.22).

## 4 Discussion

Telogen effluvium is the most common cause of non‐scarring diffuse hair loss in women [1]. As it causes a cosmetic issue, it is a significant problem for women, both psychologically and socially [2]. The etiology of TE can be idiopathic, or various factors such as endocrine disorders, medications, trauma, and emotional and physiological stress may be responsible. Therefore, a systematic approach including a detailed history, physical examination, and laboratory tests should be adopted for accurate diagnosis and early treatment in female patients presenting with hair loss complaints [5]. Iron deficiency, anemia, vitamin B12 deficiency, folic acid deficiency, and thyroid function abnormalities may be responsible for many cases [6, 7, 8]. As seen in our study, deficiencies in laboratory parameters, especially ferritin and serum iron, are commonly observed findings in women presenting with TE. However, contrary to previous studies conducted in our country, our study examined these findings in three distinct time periods—adolescence, reproductive age, and postmenopausal period—dividing women into three groups: under 18 years of age, between 18 and 45 years, and over 45 years. It was found that there were significant differences in laboratory values among women diagnosed with TE across these time periods. Ferritin, as the storage form of iron, is an important marker because it serves as a cofactor for certain enzymes involved in the hair cycle and as a building block for coenzymes; its deficiency can lead to TE [8, 9]. Ferritin deficiency was predominantly observed in the 18–45 age group, and similar to previous literature, a significant relationship (*p* = 0.041) was found. It has also been noted in the literature that ferritin deficiency in this age group may be associated with the menstrual period [9, 10]. No significant relationship was found between serum iron levels and age groups (*p* = 0.290). Similar to other studies, it was also determined that elevated ferritin levels had no significant association with TE [11]. Vitamin B12 plays a role in the development and maturation of erythrocytes, and it is known that its deficiency can disrupt this process, leading to both hair loss and graying [12, 13]. As can be seen from our study, a significant relationship was found between TE with vitamin B12 deficiency and age groups (*p* = 0.031). Folic acid plays an important role in nucleic acid and amino acid metabolism, and its deficiency can disrupt this metabolism, impair erythrocyte development, and lead to hair loss [7, 12, 14]. No statistically significant difference was found between the groups regarding folic acid deficiency (*p* = 0.603). Thyroid hormones also play an important role in hair development, and both their deficiency and excess can lead to hair loss [15, 16]. As can be understood from statistics of our study, a significant relationship was found between TE associated with thyroid dysfunction and T3 levels (*p* < 0.001), whereas no significant relationship was found with TSH levels (*p* = 0.101).

As hemoglobin is the building block of erythrocytes, it has long been used as a laboratory marker for anemia and polycythemia. As can be seen our study, no significant relationship was found between TE caused by low hemoglobin levels and age groups (*p* = 0.22), although it was more commonly observed in the 18–45 age group, which may be linked to the menstrual period, as mentioned in the literature.

The fact that ferritin is closely related with telogen effluvium (TE) but hemoglobin (Hb) does not show the same effect is due to the different physiological roles of these two proteins. Ferritin is a protein that indicates the body's iron stores and releases iron in a controlled manner, which is essential for rapidly dividing cells such as hair follicles. Hair follicles are metabolically very active in the anagen phase and require sufficient iron for essential cellular processes such as DNA synthesis. It has been reported that low ferritin levels may indicate that the iron required for these processes is insufficient and may lead to premature transition of hair follicles from anagen phase to telogen phase. This is one of the main causes of diffuse hair loss seen in TE. On the other hand, hemoglobin is a protein responsible for oxygen transport in erythrocytes and is mainly used as an indicator of anemia. However, hemoglobin cannot be considered to directly reflect the iron stores required for the metabolic processes of hair follicles. Low hemoglobin levels do not directly affect hair follicles as long as adequate oxygen can be supplied to the tissues. It can also be seen in this study that a low level of Hb (e.g., severe anemia) that could cause hair loss is generally less common in TE. Consequently, low ferritin levels are more closely associated with TE as they directly reflect the deficiency of iron required for hair follicle metabolism. In contrast, Hb levels affect oxygen transport rather than iron deficiency and can only cause hair loss in severe anemia.

## 5 Conclusion

We conclude that examining laboratory parameters in patients with telogen effluvium is important part of the systemic approach to the patient, both in terms of determining the etiology and guiding treatment and requesting these parameters based on the patient's age could be cost‐effective. At the same time, we believe that examining these laboratory parameters is an important tool in determining the etiology and guiding treatment. Moreover, laboratory tests such as vitamin B12, ferritin, and T3 are particularly important in the 18–45 age group, while folic acid deficiency is rarely seen in those under 18 or over 45, and therefore may not need to be requested for cost‐effectiveness in these cases.

## Conflicts of Interest

The authors declare no conflicts of interest.

## Data Availability

The data that support the findings of this study are available on request from the corresponding author. The data are not publicly available due to privacy or ethical restrictions.
